# A modified Austin/chevron osteotomy for treatment of hallux valgus and hallux rigidus

**DOI:** 10.1007/s10195-015-0366-7

**Published:** 2015-07-09

**Authors:** Michele Vasso, Chiara Del Regno, Antonio D’Amelio, Alfredo Schiavone Panni

**Affiliations:** Department of Medicine and Health Sciences, University of Molise, Via Francesco De Sanctis, 86100 Campobasso, Italy

**Keywords:** Austin/chevron, Modified chevron, Osteotomy, Hallux valgus, Hallux rigidus

## Abstract

**Abstract:**

The purpose of this brief paper is to present the preliminary results of a modified Austin/chevron osteotomy for treatment of hallux valgus and hallux rigidus. In this procedure, the dorsal arm of the osteotomy is performed orthogonal to the horizontal plane of the first metatarsal, the main advantage being that this allows much easier and more accurate multiplanar correction of first metatarsal deformities. From 2010 to 2013, 184 consecutive patients with symptomatic hallux valgus and 48 patients with hallux rigidus without severe metatarsophalangeal joint degeneration underwent such modified chevron osteotomy. Mean patient age was 54.9 (range 21–70) years, and mean follow-up duration was 41.7 (range 24–56) months. Ninety-three percent of patients were satisfied with the surgery. Mean American Orthopaedic Foot and Ankle Society (AOFAS) score improved from 56.6 preoperatively to 90.6 at last follow-up, and mean visual analog scale (VAS) pain score decreased from 5.7 preoperatively to 1.6 at final follow-up (*p* < 0.05). In patients treated for hallux valgus, mean hallux valgus angle decreased from 34.1° preoperatively to 6.2° at final follow-up, and mean intermetatarsal angle decreased from 18.5° preoperatively to 4.1° at final follow-up (*p* < 0.05). One patient developed postoperative transfer metatarsalgia, treated successfully with second-time percutaneous osteotomy of the minor metatarsals, whilst one patient had wound infection that resolved with systemic antibiotics.

**Level of evidence:**

Level IV.

## Introduction

Numerous corrective osteotomies have been described for surgical treatment of hallux valgus (HV), but none of them addresses all cases. One of these procedures, known for its optimal intrinsic mechanical stability, is the Austin/chevron procedure [1, 2], a V-shaped distal osteotomy, traditionally indicated for correction of mild to moderate HV, in which the hallux valgus angle (HVA) is less than 30° and the intermetatarsal angle (IMA) is less than 15° [2]. Recent studies have demonstrated that chevron osteotomy, associated with a distal soft-tissue procedure with or without the Akin procedure, can increase the amount of correction, making this combination appropriate for more severe deformities [3]. Many variants of the standard chevron have been proposed. All these variants require that the two arms of the osteotomy be oblique to the horizontal plane of the first metatarsal. However, this may generate technical difficulties and inaccuracy in executing multiplanar osteotomies, regardless of whether or not dedicated instrumentation is available.

A new modification of the chevron osteotomy was therefore proposed, requiring that the dorsal arm of the osteotomy be performed orthogonal to the horizontal plane of the first metatarsal. The main advantage is that this allows dorsal closing trapezoidal wedges to be performed much more easily and accurately, especially for correcting the proximal articular set angle (PASA), without compromising the intrinsic stability of the traditional chevron. Other advantages are the possibility of executing dorsal closing rectangular wedges, especially to obtain decompression of the metatarsophalangeal joint (MTPJ) in cases of moderate hallux rigidus (HR) [4], and of fixing the final osteotomy with a single screw.

## Materials and methods

### Surgical technique

A medial longitudinal incision is deepened to the capsule of the first MTPJ. The capsule is carefully dissected from the head of the metatarsal and base of the proximal phalanx, allowing adequate visualization of the joint. The dorsal and plantar vascular bundles are isolated and preserved. The soft-tissue rebalancing involves release of the adductor tendon from its insertion along the lateral base of the proximal phalanx, release of the deep transverse metatarsal ligament, and mobilization with recentering of the sesamoids. The medial eminence is then removed.

**Fig. 1:**
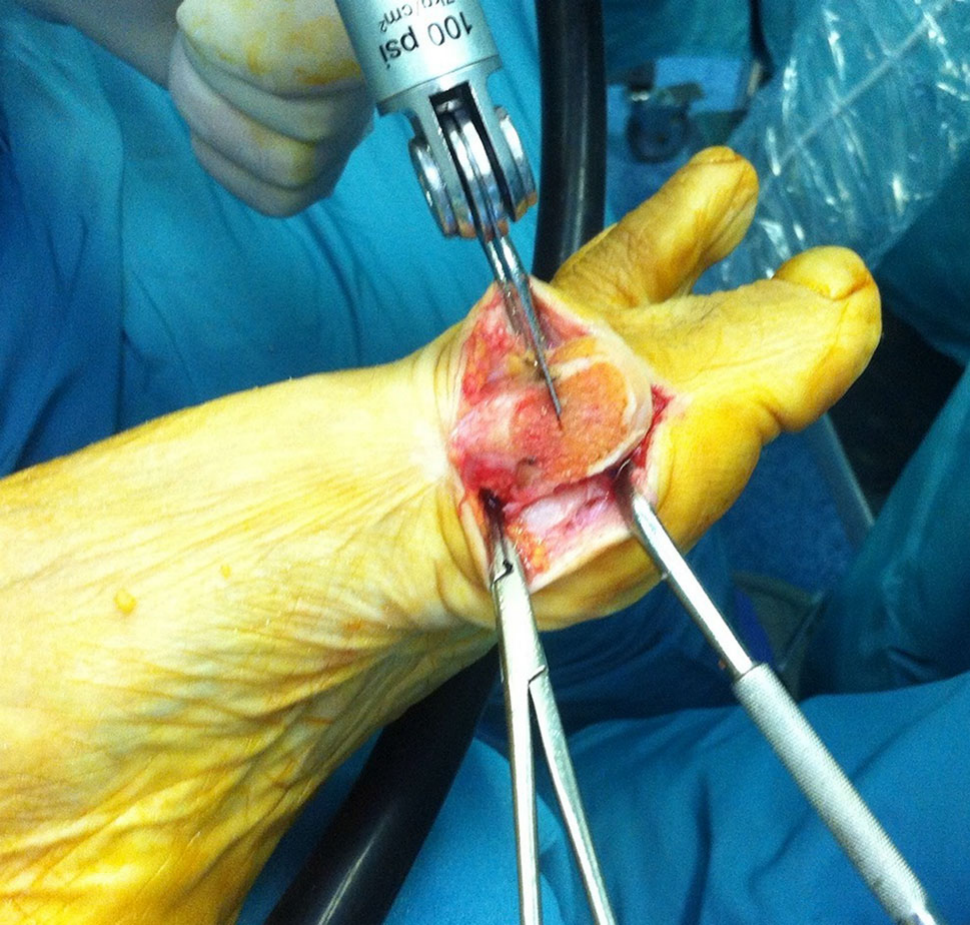
The dorsal arm of the osteotomy is performed orthogonal to the horizontal plane of the first metatarsal (approximately parallel to the MTPJ surface)

**Fig. 2:**
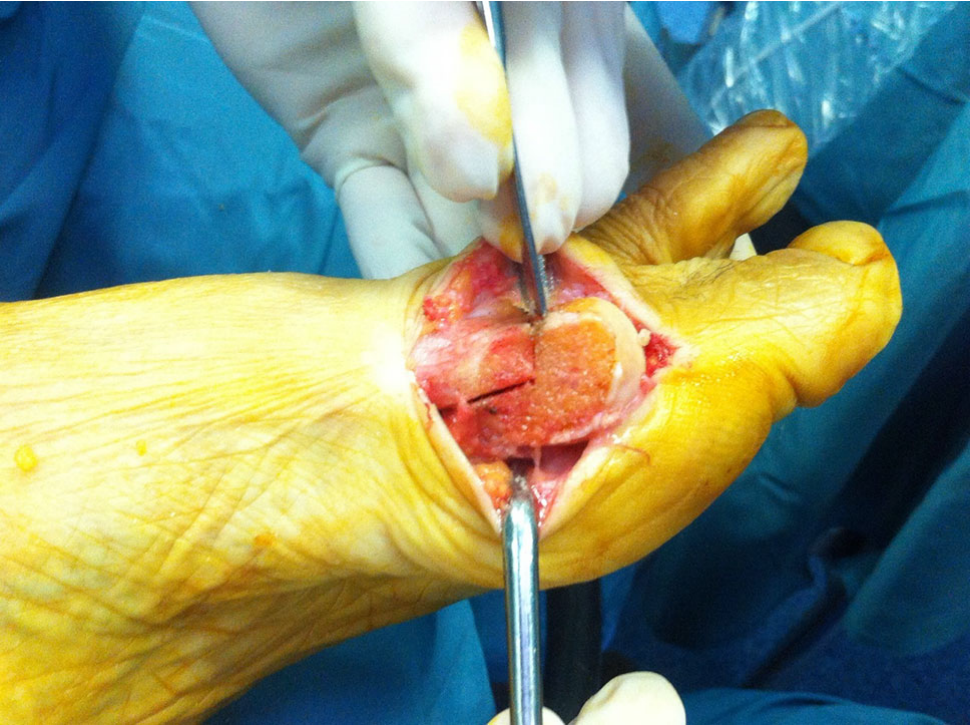
The plantar arm of the osteotomy is cut proximal to the attachment of the joint capsule to preserve the plantar vascular bundle directed to the metatarsal head fragment

**Fig. 3:**
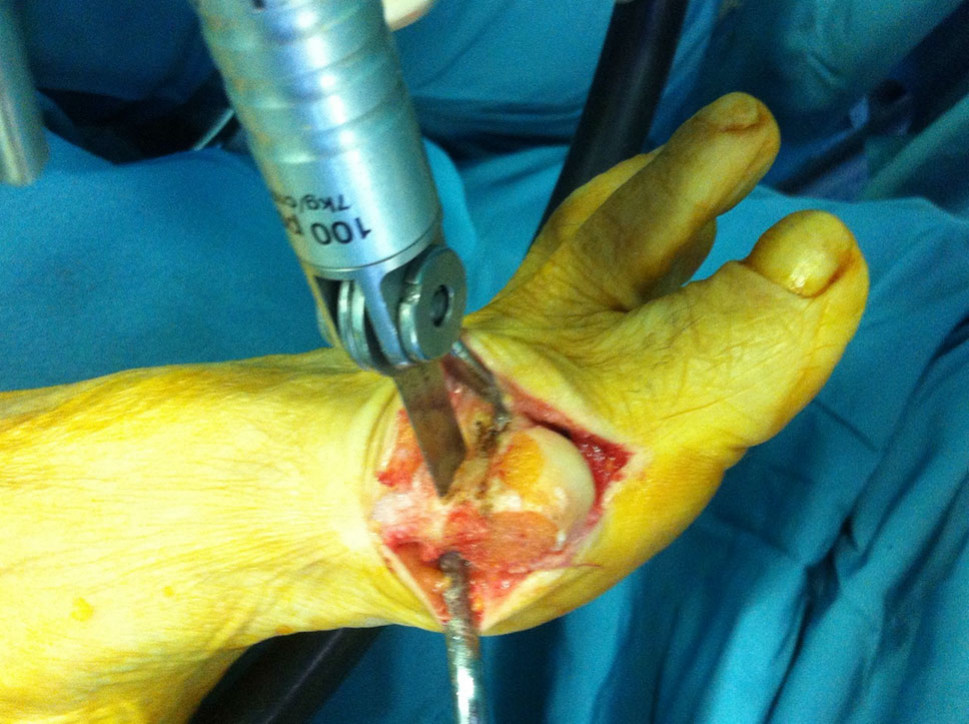
Performance of the dorsal cuts orthogonal to the horizontal plane of the first metatarsal allows one to easily obtain a precise trapezoidal wedge for accurate PASA correction

**Fig. 4:**
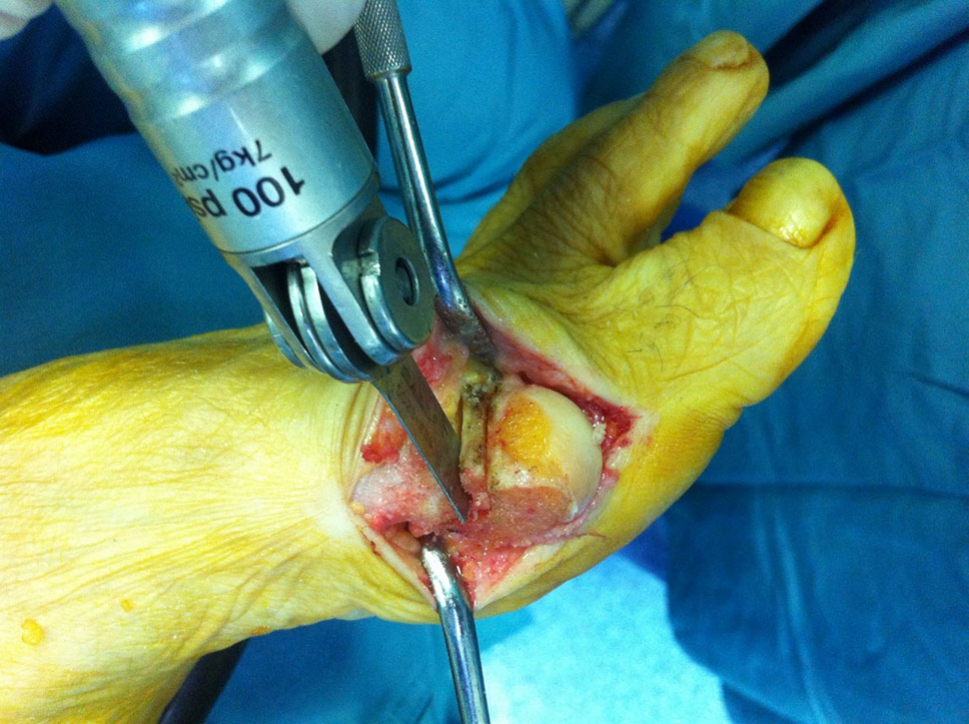
This technique allows easy performance of a precise rectangular wedge according to the desired shortening of the first metatarsal

**Fig. 5:**
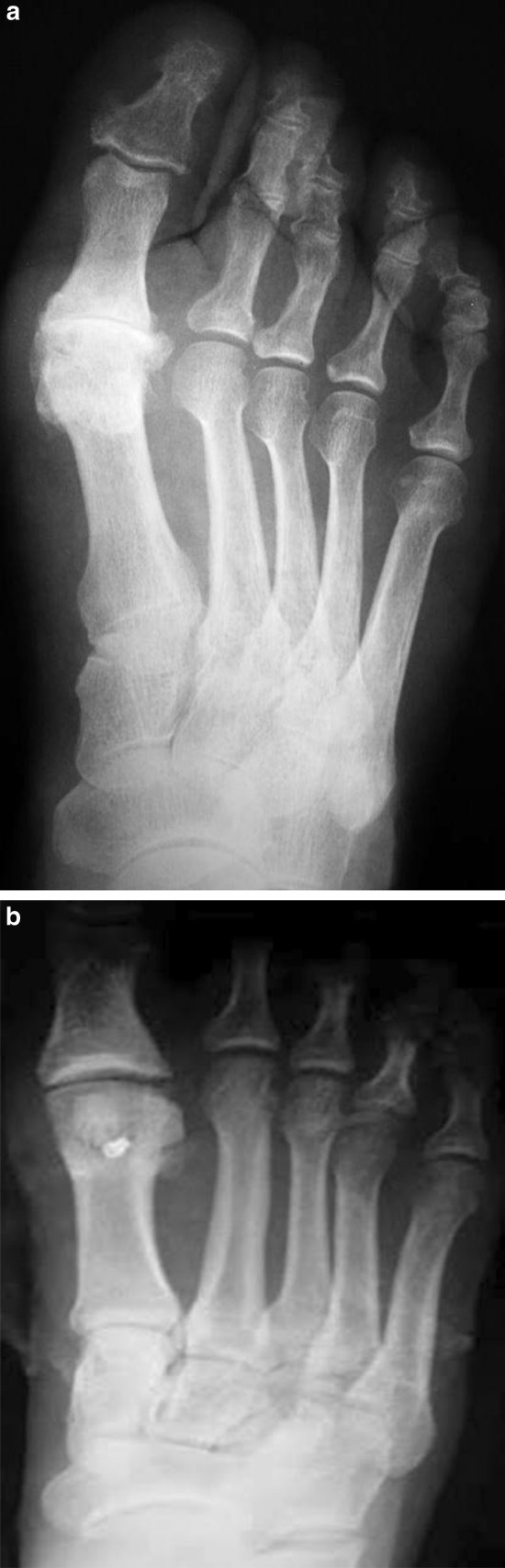
Shortened first metatarsal in hallux rigidus for MTPJ decompression. In this case, a trapezoidal wedge was necessary for PASA correction, without shifting the distal fragment laterally (therefore not affecting the IMA)

For the modified chevron, the apex of the osteotomy should remain in the center of the metatarsal head, positioned 5–10 mm proximal to the MTPJ line. As mentioned above, the dorsal arm of the osteotomy is performed perpendicular to the horizontal plane of the first metatarsal (Fig. 1). The plantar oblique (long) arm of the osteotomy is cut just proximal to the capsular attachment to the metatarsal head fragment, since this carries part of the blood supply (Fig. 2). The long plantar arm of the osteotomy makes it possible to obtain greater correction of the IMA, with the result that this technique is appropriate for more severe deformities too. When the PASA has to be corrected, a second osteotomy proximal to the dorsal cut is performed, also orthogonal to the horizontal plane of the first metatarsal; this osteotomy is obviously performed to obtain a trapezoidal wedge with medial basis (Fig. 3). The inclination of the second dorsal osteotomy relative to the sagittal plane of the first metatarsal depends on the preoperative PASA value, and therefore on the amount of correction that the surgeon desires to obtain. In the presence of HR without severe MTPJ degeneration, the second dorsal osteotomy is performed parallel (if no correction of the PASA is needed) and proximal to the dorsal cut, again orthogonal to the horizontal plane of the first metatarsal. The rectangular wedge is thus removed to decompress the MTPJ (Fig. 4). The amount of bone removed depends on the amount of shortening (and therefore decompression) that the surgeon desires to obtain (Fig. 5).

Following the osteotomies, the head fragment is translated laterally (when the IMA needs to be reduced) approximately one-quarter to one-half the width of the metatarsal bone. In all patients, only one screw, from medial to lateral, dorsal to plantar, and directed towards the apex of the osteotomies, is used to stabilize the fragments. MTPJ capsulorrhaphy is then performed (as part of soft-tissue balancing) after deflating the tourniquet. In HV patients, a percutaneous Akin osteotomy is always performed. No device is used to stabilize the Akin osteotomy.

### Postoperative care

A functional bandage is applied in the surgery room, with the hallux in overcorrected position in case of HV treatment. The first bandage is changed after 7 days, then every 2 weeks until its definitive removal, usually at 5 weeks postoperatively. Full weight bearing is allowed immediately using a talus orthopedic shoe for 5 weeks, which is generally sufficient time to obtain complete consolidation of the osteotomies. Patients treated for HR are encouraged to begin passive motion of the hallux in the immediate postoperative period. No crutches or other devices are generally necessary.

### Methods

From 2010 to 2013, the modified chevron osteotomy was performed in 184 patients with symptomatic HV and in 48 patients with HR without severe MTPJ degeneration. Patients with posttraumatic HV, previous failed HV surgery or infection, Charcot arthropathy, or severe MTPJ degeneration were excluded from this cohort. There were 189 women and 43 men. Mean patient age was 54.9 (range 21–70) years, and mean follow-up duration was 41.7 (range 24–56) months. All surgeries were performed by the same surgeon (M.V.). Patients were clinically assessed using the American Orthopaedic Foot and Ankle Society (AOFAS) [5] hallux score, visual analog scale (VAS) pain score, and patient self-reported subjective satisfaction. Patient subjective satisfaction was assessed with respect to pain, function, and cosmetic appearance, and the responses were graded as very satisfied, satisfied, improved, and dissatisfied. The HVA, IMA, and PASA were calculated radiographically before and after surgery in HV patients. The paired Student’s *t* test was used to compare the preoperative and postoperative outcomes. Statistical significance was accepted at *p* < 0.05.

## Results

At last follow-up, 161 (70 %) patients were very satisfied with the surgery, 54 (23 %) were satisfied, 14 (6 %) were improved, and 3 (1 %) were dissatisfied. Mean AOFAS score improved from 56.6 (range 49–64) points preoperatively to 90.6 (range 81–94) points at last follow-up (*p* = 0.027), and mean VAS pain score decreased from 5.7 (range 4–8) preoperatively to 1.6 (range 0–3) at final follow-up (*p* = 0.043). In HV patients, mean HVA decreased from 34.1° (range 14–44°) preoperatively to 6.2° (range −2° to 15°) at final follow-up (*p* = 0.036), and mean IMA decreased from 18.5° (range 10–28°) preoperatively to 4.1° (range 1–12°) at final follow-up (*p* = 0.041). Mean PASA decreased from 16.3° (range 12°–18°) preoperatively to 6.5° (range 4–9°) at final follow-up (*p* = 0.039). Detailed data for HV patients and HR patients are presented in Tables 1 and 2, respectively.

**Table 1:** Detailed data of HV patients

	Preoperatively	Final follow-up	*p*-Value
Mean AOFAS	54.7 ± 6.0	89.3 ± 6.2	0.027
Mean VAS	5.9 ± 1.1	1.8 ± 0.9	0.043
Mean HVA	34.1 ± 4.1°	6.2 ± 1.9°	0.036
Mean IMA	18.5 ± 3.2°	4.1 ± 2.9°	0.041
Mean PASA	16.3 ± 3.1°	6.5 ± 1.7°	0.039

**Table 2:** Detailed data of HR patients

	Preoperatively	Final follow-up	*p*-Value
Mean AOFAS	58.5 ± 6.3	91.9 ± 5.9	0.027
Mean VAS	5.5 ± 1.0	1.4 ± 0.7	0.043

One patient developed severe postoperative transfer metatarsalgia, treated successfully with a second-time percutaneous osteotomy of the minor metatarsals, whilst one patient had wound infection that resolved with systemic antibiotics. The three dissatisfied patients (two with preoperative HV and one with preoperative HR) claimed persistent pain; none of them requested reoperation. In particular, the two patients with preoperative HV complained of mild pain with stiffness of the first MTPJ, while the patient with preoperative HR developed mild transfer metatarsalgia under the second metatarsal head. There were no cases of delayed union or nonunion, metatarsal head necrosis, MTPJ stiffness (defined as range of movement <30°), displacement after fixation, or complex regional pain syndrome. In a patient with rheumatoid arthritis, two screws were necessary to fix the osteotomy because of poor bone stock.

## Discussion

The success of Austin/chevron osteotomy for correction of HV is well established [6]. Although introduced for correction of mild to moderate deformities, many studies have recently demonstrated that chevron osteotomy, associated with a soft-tissue procedure with or without the Akin procedure, can allow correction of more severe deformities [7].

Multiplanar osteotomies of the first metatarsal are indicated to correct each component of the deformity, and to avoid recurrence of the deformity itself or a noncongruent joint leading to arthritis [8, 9]. However, even when dedicated instrumentation is available, multiplanar osteotomies can prove to be technically demanding and ultimately inaccurate, with the result that oblique cut orientation may make articular fragment displacement and repositioning quite difficult [9].

The presented technique was, instead, found to be technically simple and highly reproducible, especially in the presence of multiplanar osteotomies, because the geometry of the osteotomy makes corrections easy and precise, and allows considerable versatility. Performing the dorsal arm of the osteotomy orthogonal to the horizontal plane of the first metatarsal makes it easier to obtain accurate trapezoidal or rectangular bone wedges to correct the PASA and/or decompress the MTPJ. Compensation for structural deformity in three planes is possible; in fact, the procedure may be combined with an Akin osteotomy in cases of significant interphalangeal valgus and/or residual pronation deformity. Therefore, this modified chevron procedure is effective and appropriate for correction of mild to severe HV deformities. Finally, the shape of this osteotomy does not affect the intrinsic stability of the traditional chevron, as sufficient stability is provided by impaction of the cancellous head fragment on the shaft. Additionally, unlike traditional osteotomies, suggested in cases needing MTPJ decompression for HR [9, 10], this technique requires a single screw instead of two, as reported by other authors [11].

The present modification of the standard Austin/chevron osteotomy allows easy and reproducible multiplanar correction of HV deformities, with high patient satisfaction, even in the presence of severe deformities. This procedure is also chosen in cases of HR with mild arthritis to decompress the MTPJ. In all these cases, only one screw is needed to fix the osteotomy.

## References

[1] Robinson AH, Limbers JP (2005). Modern concepts in the treatment of hallux valgus. J Bone Joint Surg Br.

[2] Fakoor M, Sarafan N, Mohammadhoseini P (2014). Comparison of clinical outcomes of scarf and chevron osteotomies and the McBride procedure in the treatment of hallux valgus deformity. Arch Bone Jt Surg.

[3] Ferrao PN, Saragas NP (2014). Rotational and opening wedge basal osteotomies. Foot Ankle Clin.

[4] Giannini S, Ceccarelli F, Faldini C, Bevoni R, Grandi G, Vannini F (2004) What’s new in surgical options for hallux rigidus? J Bone Joint Surg Am 86-A(Suppl 2):72–83 10.2106/00004623-200412002-00011 15691111

[5] Kitaoka HB, Alexander IJ, Adelaar RS, Nunley JA, Myerson MS, Sanders M (1994). Clinical rating systems for the ankle-hindfoot, midfoot, hallux, lesser toes. Foot Ankle Int.

[6] Trnka HJ, Zembsch A, Weisauer H (1997). Modified Austin procedure for correction of hallux valgus. Foot Ankle Int.

[7] Bai LB, Lee KB, Seo CY, Song EK, Yoon TR (2010). Distal chevron osteotomy with distal soft tissue procedure for moderate to severe hallux valgus deformity. Foot Ankle Int.

[8] Nery C, Barroco R, Réssio C (2002). Biplanar chevron osteotomy. Foot Ankle Int.

[9] Freeman BL, Hardy MA (2011). Multiplanar phalangeal and metatarsal osteotomies for hallux rigidus. Clin Podiatr Med Surg.

[10] Polzer H, Polzer S, Brumann M, Mutschler W, Regauer M (2014). Hallux rigidus: joint preserving alternatives to arthrodesis—a review of the literature. World J Orthop.

[11] Dickerson JB, Green R, Green DR (2002) Long-term follow-up of the Green-Watermann osteotomy for hallux limitus. J Am Podiatr Med Assoc 92:543–554 10.7547/87507315-92-10-543 12438500

